# The nitroxyl donor isopropylamine-NONOate elicits soluble guanylyl cyclase-dependent antihypertrophic actions: comparison of the potential therapeutic advantages of HNO over NO•

**DOI:** 10.1186/2050-6511-14-S1-P56

**Published:** 2013-08-29

**Authors:** Rebecca Ritchie, Jennifer Irvine, Nga Cao, Swati Gossain, Amy Alexander, Jane Love, Chengxue Qin, John D Horowitz, Barbara K Kemp-Harper

**Affiliations:** 1 Heart Failure Pharmacology, Baker IDI Heart & Diabetes Institute, Melbourne, Vic, 8008, Australia; 2 Department of Medicine, Monash University, Clayton, Vic 3800, Australia; 3 Department of Pharmacology, Monash University, Clayton, Vic 3800, Australia; 4 The Queen Elizabeth Hospital, Woodville, SA 5011, Australia

## Background

Nitroxyl (HNO) is a redox congener of NO•. We now directly compare the antihypertrophic efficacy of HNO and NO• donors in cardiomyocytes *in vitro*, and compare their contributing mechanisms of actions in this setting. In addition, the ability of the HNO donor isopropylamine-NONOate to acutely limit hypertrophic responses in the intact, beating rat heart was also determined.

## Results

IPA-NO elicited concentration-dependent inhibition of endothelin-1 (ET_1_)-induced increases in neonatal rat cardiomyocyte size, with similar suppression of hypertrophic genes. Antihypertrophic IPA-NO actions were significantly attenuated by L-cysteine (HNO scavenger), Rp-8-pCTP-cGMPS (cGMP-dependent protein kinase inhibitor), and ODQ (to target soluble guanylyl cyclase, sGC), but were unaffected by carboxy-PTIO (NO• scavenger) or CGRP_8-37_ (calcitonin gene-related peptide antagonist). Furthermore, IPA-NO significantly increased cardiomyocyte cGMP 3.5-fold (an L-cysteine-sensitive effect), and stimulated sGC activity 3-fold, without detectable NO• release. IPA-NO also suppressed ET_1_-induced cardiomyocyte superoxide generation. The pure NO• donor, DEA-NO, reproduced these IPA-NO actions, but were sensitive to carboxy-PTIO rather than L-cysteine. Although IPA-NO stimulation of purified sGC was preserved under pyrogallol oxidant stress (in direct contrast to DEA-NO), cardiomyocyte sGC activity after either donor were attenuated by this stress. Excitingly IPA-NO also exhibited acute antihypertrophic actions in response to acute pressure-overload in the intact heart, at doses that did not affect coronary perfusion pressure or LV function.

## Conclusion

Our results demonstrate that the antihypertrophic actions of HNO are broad, and not donor-specific, and are likely independent of its vasodilator and other hemodynamic actions. Given that HNO is impervious to reactivity with superoxide, HNO donors may offer therapeutic advantages over NO• donors. These studies further strengthen the therapeutic promise of HNO donors and other non-nitrovasodilator means of cGMP-elevation as a novel strategy for cardiac pathologies, as stand-alone and/or add-on therapy to standard care.

